# Partial Characterization of Lectins Purified from the Surco and Vara (Furrow and Rod) Varieties of Black *Phaseolus vulgaris*

**DOI:** 10.3390/molecules27238436

**Published:** 2022-12-02

**Authors:** Carmen Valadez-Vega, Olivia Lugo-Magaña, Gabriel Betanzos-Cabrera, José Roberto Villagómez-Ibarra

**Affiliations:** 1 Área Académica de Medicina, Instituto de Ciencias de la Salud, Universidad Autónoma del Estado de Hidalgo, Ex-Hacienda de la Concepción, Tilcuautla, San Agustín Tlaxiaca 42080, Mexico; 2 Preparatoria Número 1, Universidad Autónoma del Estado de Hidalgo, Av. Benito Juárez S/N, Constitución, Pachuca de Soto 42060, Mexico; 3 Área Académica de Nutrición, Instituto de Ciencias de la Salud, Universidad Autónoma del Estado de Hidalgo, Ex-Hacienda de la Concepción, Tilcuautla, San Agustín Tlaxiaca 42080, Mexico; 4 Área Académica de Química, Instituto de Ciencias Básicas e Ingeniería, Universidad Autónoma del Estado del Hidalgo, Ciudad del Conocimiento, Mineral de la Reforma 42184, Mexico

**Keywords:** lectin, erythrocytes, functional component, antinutritional

## Abstract

As they manifest specifically and reversibly, lectins are proteins or glycoproteins with the characteristic of agglutinating erythrocytes. Given that grain legume lectins can represent 10% of protein content and can have various biological functions, they are extensively studied. The objective of this work was to purify and partially characterize the lectins of *Phaseolus vulgaris* black, var surco and vara (LBBS and LBBV). Both lectin types were purified by affinity chromatography on stroma matrix, which agglutinated human erythrocytes type A, B, and O, as well as rabbit, hamster, pig, and chicken erythrocytes. Native-PAGE was employed for molecular mass determination, yielding 109.36 and 112.68 kDa for BBS and BBV, respectively. Further analyses revealed that these lectins are tetrameric glycoproteins that require Ca^+2^, Mn^+2^ and Mg^+2^ ions for exhibiting their hemagglutinating function, which can be inhibited by fetuin. Moreover, optimal pH was established for both lectins (10.5 for LBBS and 7−9 for LBBV), while their activity was temperature-dependent and ceased above 70 °C. Finally, the observed differences in the biochemical characteristics and bioactive functions were ascribed to the different physiological characteristics of each seed, as well as the protein itself.

## 1. Introduction

In several Latin American countries, beans are widely cultivated and consumed due to their high content of protein, carbohydrates, fiber, vitamins, and minerals [1].

Of the 150 bean species known around the world, four species are cultivated and consumed in Mexico: common bean, comba, ayocote, and tepari, which have different colorations; consumption varies between regions of the country. One of the varieties consumed in Mexico is the black bean, and there is great variety in black beans [2]. Despite being a food with favorable nutritional benefits, it has been shown that this food contains some compounds that are considered to be anti-nutritional, such as tannins, phytates, protease inhibitors, and lectins that can affect the bioavailability and digestibility of nutrients [3].

Legume lectins have been extensively studied because they are toxic proteins that represent a health risk, as some are capable of resisting the gastrointestinal digestion process; in legume seeds, they can represent up to 10% of the total protein in the mature seed [4,5].

Plant lectins, ubiquitously distributed in a variety of plant species, are carbohydrate-binding proteins of non-immune origin. They are extensively studied because they are toxic proteins that represent a health risk, as some are capable of resisting the gastrointestinal digestion process [5]. Moreover, they are a valuable tool in the study of glycoproteins and cell membrane oligosaccharides and can be used in cell identification and separation, histochemistry, cytochemistry, neuronal pathway mapping, mitogenic stimulation of lymphocytes, bone marrow purging for transplantation, selection of lectin resistant mutants, glycoprotein biosynthesis, and many other contexts.

This research interest is also motivated by their important biological functions as their anticarcinogenic, antifungal, antimicrobial, immunomodulatory, and immunomodulatory properties [4,6,7,8,9] make them suitable candidates for cancer therapy and transplantation and other potential applications [10,11].

As lectins are present in all living organisms, they are studied in viruses, bacteria, plants, and animals [12,13]. In the plant kingdom, these proteins are found in all parts of plants, where they are posited to provide defense against insects and pathogens, as well as facilitate protein storage, seed dormancy maintenance, carbohydrate transport, and symbiosis, among other physiological functions [14,15,16].

Lectins have a wide range of specificity, binding to either monosaccharides or complex carbohydrates, binding in a non-covalent way across hydrogen bounds (hydrogen bounds) electrostatic interactions, and hydrophobic stacking. They are frequently utilized when studying the structure of carbohydrates in cells and purification of glycosylated molecules in order to expand the use of these proteins [15,17,18]. The characteristics of lectins can be very varied, even within the same genus or species, as is the case of *Phaseolus vulgaris*, in which lectins can vary in content, number of subunits, carbohydrate binding specificity, amino acid sequence in their isoforms, and biological activity.

In this context, lectins of the genus *Phaseolus* are of particular value, as indicated by a considerable body of glycobiology research focusing on the purification, characterization, and functions of lectins from various species of beans, such as *Phaseolus vulgaris*, *Phaseolus acutifolius*, *Phaseolus coccineus,* and *Phaseolus lunatus*. These studies indicate that these lectins present isoforms and are glycosylated tetrameric proteins that can be used for obtaining bioactive peptides but require metals for their biological function. Moreover, they consist of two polypeptide chains L and E, which indicate their binding to leukocytes and erythrocytes, respectively; they may exist in five possible tetrameric isoforms (E4, E3L1, E2L2, E1L3, and L4) [18,19] as well as stimulate mitogenesis and can act as both antiviral and antimicrobial agents. As they are cytotoxic to various cell types, they can exhibit adverse toxicological effects, which may result in death [6,9,13,20,21,22,23,24,25,26,27,28].

Despite these beneficial findings, further research is still needed, as lectins from different sources can have important structural and functional differences [29]. Although many lectins from different bean species are known, it is convenient to continue studying these proteins from this type of seed, since there may be functional and structural differences that may allow us to broaden our knowledge and possible uses.

Thus, to contribute to this ongoing endeavor, as a part of the present study, lectins from the surco (BBS) and vara (BBV) varieties of black bean *Phaseolus vulgaris* were purified to facilitate their partial characterization.

## 2. Results

### 2.1. Protein Content

The Kjeldahl method was adopted to determine the total protein content, yielding 21% for BBV and 17% for BBS, as shown in Table 1 along with the corresponding crude extract values.

### 2.2. Lectin Purification

**Figure 1:**
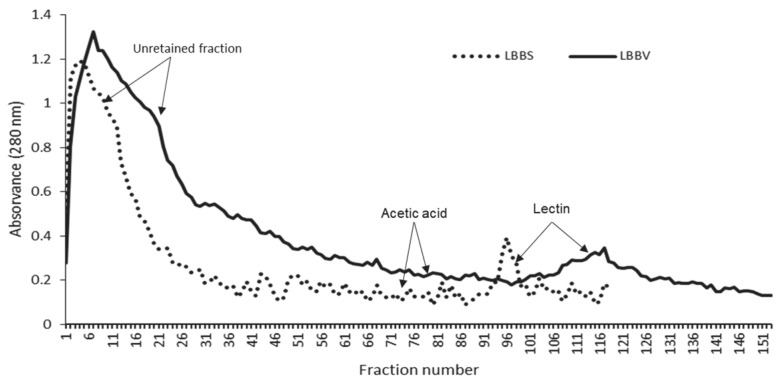
Black bean surco (BBS) and black bean vara (BBV) lectin purification in an affinity column containing stroma immobilized on Sephadex G25. Protein aqueous extracts of BBS and BBV were applied onto affinity column; the unretained fraction was eluted with PBS (50 mM), and the retained fraction, corresponding to lectin, was eluted with acetic acid (3%).

For both BBV and BBS, lectin purification was carried out in an affinity column containing stroma immobilized on Sephadex G25. The chromatograms obtained after elution with acetic acid (in which most of the hemagglutinating activity was recovered) showed only one peak corresponding to lectin. As shown in Figure 1, lectins were collected in the ranges 88−101 and 97−127, for BBS and BBV, respectively.

The extracts of both beans were characterized by high soluble protein concentration and low lectin activity, as shown in Table 1. After purification, lectin activity increased by 1,115,170% for BBV and 7.5% for BBS with respect to the crude extract.

### 2.3. Polyacrylamide Gel Eletrophoresis (SDS-PAGE and NATIVE-PAGE)

**Figure 2:**
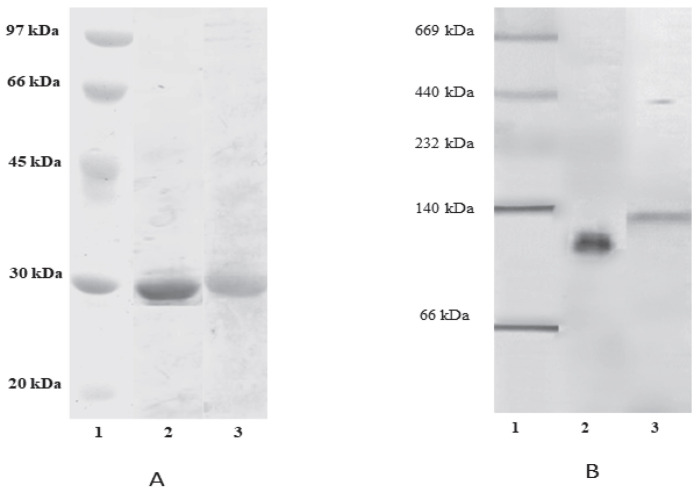
(**A**) SDS-PAGE of purified BBV and BBS lectins; (**B**) corresponding NATIVE-PAGE results. Notes: 1−Molecular weight marker, 2−Pure lectin from the vara black bean variety, 3−Pure lectin from the surco black bean variety.

The electrophoretic patterns of purified lectins shown in Figure 2 indicate that both lectins produce one band in the retained fraction. According to the SDS-PAGE electrophoretic pattern depicted in Figure 2A, both lectins were purified correctly, resulting in 27.34 and 28.17 kDa molecular weight (MW) for lectin black been vara (LBBV) and lectin black been surco (LBBS), respectively. The electrophoretic pattern under native conditions presented in Figure 2B shows a single band corresponding to the purified lectin, whereby the differences between SDS-PAGE and NATIVE-PAGE findings are attributed to the three-dimensional structure of the lectins.

### 2.4. Hemagglutination Activity

The hemagglutinating activity of both purified lectins was determined in trypsinized erythrocytes, of which, three were of human origin, and six were derived from animal species. Table 2 shows the hemagglutination titers of both lectins, indicating that the proteins have a broad spectrum of binding to human and animal erythrocytes; both lectins were statistically different (*p* < 0.05). The effect of the two lectins BBV and BBS on hemagglutinating activity was evaluated in two groups, human erythrocytes (A, B, and O) and animal erythrocytes (rabbit, chicken, hamster, beef, pig, and sheep). It was found that, for BBV in A and B erythrocytes, there is no difference. In BBS, all blood types are different. For the two lectins studied in animal erythrocytes, all show significant differences.

Moreover, higher hemagglutination titers were noted for LBBS in all types of erythrocytes, indicating its preference for type O erythrocytes. Nonetheless, the observed affinity for type O human erythrocytes was considerably exceeded by hamster erythrocytes (which was 1.8 × 10^16^ greater), while the lowest affinity was noted for rabbit and bull erythrocytes.

On the other hand, LBBV exhibited the highest affinity for hamster erythrocytes, followed by rabbit and chicken and finally human erythrocytes, albeit with equal affinity for type A and B and very low affinity for type O erythrocytes.

### 2.5. Carbohydrate and Mineral Content

Carbohydrate content analysis, which yielded 11.39% and 9.54% for LBBS and LBBV, respectively, indicated that both lectins are glycoproteins.

Moreover, plasma spectrometry results shown in Table 3 indicate that Ca^+2^ and Mg^+2^ are present in higher concentration relative to Mn^+2^ and Cu^−1^. While Ca^+2^, Mn^+2^, and Zn^+2^ concentrations in BBS were 3.16−37.12% higher than in LBBV, Cu^−1^ and Mn^+2^ content was, respectively, 22.59% and 21.21% higher in LBBV relative to LBBS.

EDTA treatment significantly reduced (*p* < 0.05) the ion content in both lectins, with the greatest decrease noted for Mg^+2^ and Mn^+2^ ions (17.41−52%). On the other hand, in LBBS, the greatest decline was noted for Mg^+2^ and Ca^+2^ (77.92% and 75.82%, respectively), while the effect on Mn^+2^ was minimal. As can be seen from Table 4, these decrements are directly related with the decrease in the hemagglutinating activity (to 1.49 × 10^−6^% of the original level). Once some of the ions were reconstituted, the biological activity was recovered and even surpassed the values obtained for the native lectin, whereby the greatest effect was exerted by Mg^+2^, followed by Ca^+2^, and finally Mn^+2^.

### 2.6. Inhibition of Hemaglutinating Activity

In this work, the inhibitory effect of monosaccharides, oligosaccharides, and glycoproteins on the hemagglutinating capacity of lectins was also studied, and the obtained results indicate that fetuin caused hemagglutination inhibition in both lectins. As can be seen from Table 5, LBBS was also inhibited by monosaccharide mannose, while trisaccharide raffinose was the only other inhibitor of LBBV.

### 2.7. Influence of pH and Temperature on Hemaglutinating Activity

**Figure 3:**
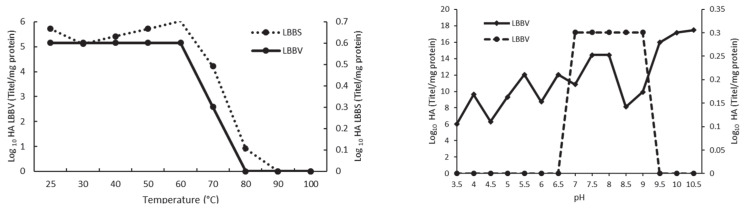
The influence of pH and temperature on the hemagglutinating activity of lectins purified from surco and vara black beans varieties.

The two studied black bean lectins exhibited significant differences in their hemagglutinating activity as a function of pH. The results reported in Figure 3 show that, in the 3.5−6.5 and 9.5−10.5 pH ranges, LBBV lost its biological activity, while maintaining some of its activity in the 7−9 pH range. On the other hand, LBBS was very stable irrespective of the pH values and maintained strong activity throughout the analyzed pH range. Moreover, numerous dilutions were required to reduce its erythrocyte hemagglutination capacity (the greatest decrease was achieved at the 3.5, 4.5, and 8.5 pH values). Conversely, the highest titers were obtained at pH 9.5, 10, and 10.5, with dilutions reaching 2^53^, 2^57^, and 2^58^, respectively. Similar results to those related to LBBV and LBBS have been reported for several bean lectins, which have shown stability in the 0−70 °C temperature range.

Data shown represent mean ± SEM based on the results obtained in at least three independent experiments.

## 3. Discussion

In this study, partial characterization of two lectins purified from the *Phaseolus vulgaris* variety surco and *Phaseolus vulgaris* variety vara, respectively, was performed as these varieties are commonly found in a typical Mexican diet. For this purpose, total proteins were first determined in crude extract, and the obtained results concurred with those reported for other varieties such as black turtle bean, belonging to the P. vulgaris family (19.54−22.62%) [30], or for the common black bean (23.9 and 25.9%). According to the available data, the differences in nutrient content (including protein) may be due to the variations in climate, as rainfall and temperature, as well as timing and area of cultivation have been shown to impact these parameters [31,32].

In practice, affinity chromatography is typically employed for lectin purification, and this strategy yielded 43.7% higher recovery for BBV relative to BBS in our analyses, demonstrating that this column is more suited to purifying lectins from BBV.

In the present study, a stroma column obtained from erythrocytes was utilized in affinity chromatography, as this is also a standard approach and has previously allowed most of the activity to be recovered from lectin purified from *Phaseolus acutifolius* in the fraction eluted with glycine-HCl, which resulted in a 347-fold increase in hemagglutination. This type of column has also been used in the purification of lectin from *Amaranthus cruentus* and *Amaranthus leucocarpus*. However, these results differ significantly from those obtained for the surco and vara black beans varieties, possibly due to the differences in the purification sources, the affinity they exhibit towards the chosen matrix, and the purification conditions [33,34].

The chromatographic patterns obtained for both surco and vara varieties differ from those reported for other types of *Phaseolus vulgaris*, such as Anasazi bean, which was previously purified by affi-gel blue column and ion exchange chromatography [17], as well as common black bean purified by a reverse micellar system [26] and by affinity chromatography on agarose-fetuin column [9].

The purification results were further utilized in this work for determining protein sizes by NATIVE-PAGE and SDS-PAGE methods, given that several *Phaseolus* lectins are composed of four subunits [35,36]. Available data further indicate that the MW of *Phaseolus vulgaris* lectin subunits can vary between 30 and 36.5 kDa [6,9,24,25,37], concurring with the values obtained for LBBV and LBBS using SDS-PAGE.

The molecular weights yielded by NATIVE-PAGE were approximately four times higher than those obtained using SDS-PAGE. This difference aligns with the results reported for several *Phaseolus* species, whose MW ranged from 21 to 35 kDa and which were found to exhibit homotetrameric structure [6,9,13,22,23,38,39,40]. Therefore, the results obtained for the vara and surco bean lectins indicate that these lectins likely have this type of tetrameric structure consisting of identical subunits, as this assumption is supported by extant evidence pertaining to several bean lectins which have this type of structure, with MW in the 83−150 kDa range [6,36,38,41].

Lectin hemagglutination was also evaluated in this work in order to establish whether they show specificity towards any type of erythrocytes, revealing higher hemagglutination titers for LBBS in all types of erythrocytes. Moreover, this lectin showed preference for type O erythrocytes, followed by α-D-Galactose and α-D-N-Acetyl Galactosamine found in erythrocytes B and A, respectively [4,9,14,42]. Rabbit erythrocytes are widely used in lectin hemagglutination activity tests, since many of these proteins tend to react preferentially with erythrocytes from this source [9], as demonstrated for lectins derived from *Erythrina speciosa*, *Andira anthelmia*, *Phaseolus vulgaris, Phaseolus coccineous,* and *Moringa oleifera* [23,43,44,45,46].

Erythrocytes from various animal sources such as sheep, horse, cow, guinea pig, rat, mouse, and chicken have also been used in extant research to evaluate the hemagglutinating activity of lectins from *Erythrina speciosa*, *A. naeslundii*, *Phaseolus vulgaris,* and *Phaseolus coccineus* [23,43,44,45,46], revealing a wide spectrum of biological activity for different erythrocyte sources, thus supporting the current findings.

Likewise, the findings related to carbohydrate content are in agreement with those reported for lectins of the genus *Phaseolus*, such as *P. vulgaris*, *P. coccineousu,* and *P. acutifolius* (5.98−7.56%) [6,9,36,38,41,47,48]. Many legume lectins are recognized as metalloproteins, containing Ca^+2^, Mn^+2^, Mg^+2^, and other ions important for their biochemical activity. Thus, eliminating them causes a decrease or loss of hemagglutinating activity [49,50].

Lectins such as those from *Phaseolus coccineous*, *Phaseolus acutifolius*, *Inocybe umbrinella*, LBBV, and LBBS, which contain higher Ca^+2^ concentrations, are recognized as C-type lectins [9,38,51]. Ions such as Ca^+2^ and Mn^+2^ are attached to each subunit located in the vicinity of the carbohydrate binding site, and, thus, participate in the stabilization and specificity of the biological activity of the lectin, since these ions interact with water and carbohydrates to which they bind [14,52,53,54].

EDTA treatment caused a reduction in the ion content of the lectins studied in this work, which suggests that ions are strongly bound to the protein fraction of the lectin [9].

An ample body of research indicates that lectin demetallization induces conformational changes in the regions in which metals are found, causing alterations in the lectins’ ability to bind carbohydrates, as reflected in the decrease or loss of their biological activity. However, some aspects of the secondary structure, such as the lamellae and helices, do not exhibit severe alterations as a result of stability imparted by hydrogen bridges without causing alterations in the quaternary structure [53,55,56]. In the present study, reconstitution of some of the metal ions allowed LBBV and LBBS biological activity to be recovered, and even exceed that of the native lectin, whereby Mg^+2^ caused the greatest effect, followed by Ca^+2^, and finally Mn^+2^. These findings are in line with those reported by other authors [53,57]. For example, when Ca^+2^, Mg^+2^, and Mn^+2^ are chelated from lectins, hemagglutination is inhibited, whereas Fe^+2^, Na^+1^, Ba^+2^, and K^+1^ ions do not cause this effect [58,59]. The hemagglutination tests in demetallized lectins after the addition of Ca^+2^, Mg^+2^ and Mn^+2^ conducted as a part of this investigation further showed that these ions are essential for both lectins to maintain their biological function, with the effect of Mg^+2^ exceeding that of Ca^+2^ and Mn^+2^ by 96−99%. Moreover, inhibition of hemagglutinating activity revealed that both lectins have an affinity for complex carbohydrates, such as those found in glycoproteins. These results are in agreement with the findings reported for lectins from tepary, ayocote, lima, or common bean, indicating that lectin activity is unaffected by simple sugars, while it is inhibited by complex oligosaccharides such as thyroglobulin, ovalbumin, and fibrinogen [6,9,17,22,60].

Although some lectins show affinity for some monosaccharides [37,58], their affinity for oligosaccharides or complex glycans is higher, as lectins contain some amino acid residues that participate in the binding with other residues that make up the oligosaccharide [14]. Finally, studies in which the influence of pH and temperature on hemagglutinating activity was evaluated indicate that some lectins (including LBBV as well as lectins from *Parkia panurensis* and *Dolichos lablab*) only maintain their activity in near-neutral pH ranges [50,61].

These alterations in lectin activity due to changes in pH are attributed to ionization of the amino acid side chains, which modifies the binding capacity of the protein to carbohydrates on the erythrocyte surface, leading to a decrease or loss of hemagglutination, as these changes in the amino acid residues of lectins may cause protein denaturation [62,63].

When temperature effects on LBBV and LBBS were examined, their stability in the 0−70 °C range was confirmed, concurring with the results obtained in other studies focusing on other bean lectins. On the other hand, higher temperatures have been found to cause considerable decrease in hemagglutinating activity, which typically ceases above 90 °C [17,60,61,64,65,66]. Moreover, as the hemagglutinating activity of lectins is dependent on their native tridimensional structure, any structural changes would impact the biological activity of the protein, ultimately causing denaturation and, therefore, complete loss of hemagglutinating capacity [13,67,68].

In extant studies, oligosaccharides have been found to improve the stability of glycoproteins when exposed to physicochemical treatments, and this phenomenon has been attributed to the fact that glycans increase the electrostatic interactions of proteins, helping preserve their structure. It has also been reported that these interactions stabilize the native structure, since the number of non-covalent bonds increases, which provides stability against thermal damage and various chemical agents. Likewise, the presence of intramolecular disulfide bridges in the protein was posited by several authors to impart greater thermal stability to some proteins [16,63,69].

These and similar findings reported in extant literature may explain the differences observed between the two lectins in the focus of the present research, since LBBS contained a greater amount of carbohydrates in its structure and was less influenced by pH and temperature compared to LBBV. These results also concur with those reported for *Phaseolus coccineous* lectin, where the variety with the highest carbohydrate content also exhibited the greatest thermal stability [9].

## 4. Materials and Methods

### 4.1. Plant Material

The two black bean varieties—black bean variety vara (BBV) and black bean variety surco (BBS)—required for this investigation were obtained from the Huasteca Hidalguense, in Huejutla, Hidalgo, Mexico (21°10′25.6″N 98°21′47.7″W). The purchased seeds were cleaned before being ground in a grain mill (Analitycalv mill, 4301-00, Cole Palmer, IL, USA) and sieved through a 40-mesh (USA Standard Testing). The resulting flour was packed in airtight bags and refrigerated at 4 °C until required for analyses.

### 4.2. Chemicals

Standard multi-ionic solution for plasma spectrometry was obtained from Perkin-Elmer (Waltham, MA, USA), while trypsin, molecular weight markers, and glycine were acquired from Sigma Chemical Co. (St Louis, MO, USA). All other chemicals were of analytical grade.

### 4.3. Protein Quantification

Been seed flour was analyzed according to the 920.87−1920 AOAC method using a factor of 6.25 to calculate the percentage of total protein. Bradford’s method was employed for protein quantification in crude extract and during lectin purification, with bovine serum albumin (BSA) serving as the standard. The relative protein concentration of the eluted fractions was determined by measuring the absorbance at λ = 280 nm [70,71].

### 4.4. Lectin Extraction

Lectin extraction was performed using the methodology described by Valadez-Vega and colleagues [6]. Briefly, prior to analyses, the bean meal was suspended in 10 mM phosphate-salt buffer solution (PBS 1:10 *w*/*v*, pH 7.4), and was shaken for 16 h at 4 °C. Next, proteins were precipitated with 70% (NH_4_)_2_SO_4_ from the supernatant obtained by centrifugation, and precipitate was subsequently dialyzed against PBS before being used for lectin purification.

### 4.5. Lectin Purification

The affinity column used for lectin purification was prepared with human erythrocyte stroma matrix, according to the methodology described by Zenteno and colleagues [34]. Briefly, stroma was obtained by lysis of human type A erythrocytes, washed with deionized H_2_O, and fixed with 2% glutaraldehyde overnight under refrigeration, after which it was washed with deionized water. The resulting sample was kept overnight in 1 M glycine solution at 4 °C and was subsequently washed with NaCl (0.9%). The obtained stroma was mixed with Sephadex G25 (Amersham Biosciences PD-10, Piscataway NJ, USA) and was placed in a glass chromatographic column (2.5 × 30 cm). Prior to use, the column was equilibrated with 50 mM PBS pH 7.4 after which 10 mL of protein extract was applied to the column, and the non-retained fraction was washed with PBS at a flow rate of 3 mL/min. The retained lectin fraction was eluted with 3% acetic acid and was monitored spectrophotometrically at λ = 280 nm (Perkin Elmer Lambda 40 UV/Vis spectrometer, Waltham MA, USA). The recovered lectin was dialyzed for 24 h against deionized water with three changes and was subsequently lyophilized and stored at −20 °C until use.

### 4.6. Hemagglutinating Activity

Human erythrocytes type A, O, and B from healthy donors who signed informed consent, as well as erythrocytes from chicken, beef, rabbit, pig, hamster, and sheep were used for determining the hemagglutinating activity. For this purpose, blood was collected in tubes containing anticoagulant, and erythrocytes were obtained by centrifugation at 2500 rpm for 10 min, after which the samples were washed three times with fresh volumes of PBS. Next, a trypsin solution (0.6 mg/mL, Sigma Chemical Co., St Louis, MO, USA) was added to the erythrocyte suspension, after which the sample was incubated for 60 min at 37 °C under agitation. Finally, the trypsinized erythrocytes were washed with PBS and were resuspended (2%) in PBS until required for further analyses [6].

The hemagglutination assay was performed according to the serial dilution (2-fold) method, using 96-well (U-shaped) microtiter plates. For this purpose, 50 μL of lectin solution was added to the first well and serial dilutions were performed, adjusting the volume in each well to 50 µL with PBS, whereby 50 µL of the trypsinized erythrocyte suspension (2%) was added to each well. The reaction mixture was incubated for 60 min at room temperature, and the last dilution showing hemagglutination was observed to obtain the agglutination titer. The hemagglutination titer was defined as the reciprocal of the highest dilution showing detectable agglutination and the inverse of the last dilution showing hemagglutination [6]. The hemagglutination units (HU/mg) were calculated by dividing the hemagglutination titer by the soluble protein concentration (mg) in the sample, determined by the Bradford method [72].

### 4.7. Hemagglutination Inhibition

The ability of monosaccharides (mannose, glucose, galactose, and fructose), oligosaccharides (maltose, trehalose, and raffinose), and glycoproteins (ovalbumin and fetuin) to inhibit the hemagglutination reaction of lectins was determined.

For this purpose, each of the sugar solutions (50 mM) were serially diluted in order with PBS in 96-well (U-shaped) microplates, whereby 50 µL of lectin solution (0.1 mg/mL) was added to each well and was incubated for 60 min at room temperature, after which 50 µL of trypsinized erythrocyte suspension was added to each well. After incubation for 60 min at room temperature, the last dilution that showed hemagglutination was observed, and the lowest concentration of the sugars that inhibited hemagglutination was determined [6,9].

### 4.8. Polyacrylamide Gel Electrophoresis (PAGE)

PAGE was performed according to the Laemmli technique [73], whereby 12% gels under reducing conditions were used for electrophoresis under denaturing conditions (SDS-PAGE). Electrophoresis was performed in a vertical mini VE (Amersham Biosciences, Piscataway, NJ, USA). The molecular weight of each lectin was estimated using a mixture of molecular weight markers containing phosphorylase B, bovine albumin, ovalbumin, carbonic anhydrase, soybean trypsin inhibitor, and lactoalbumin at 97, 66, 45, 30, 20, and 14 kDa, respectively (Sigma Chemical Co., St Louis, MO, USA).

Electrophoresis on native gels (NATIVE-PAGE) was performed on 7% polyacrylamide gels in a vertical mini VE (Amersham Biosciences, Piscataway NJ, USA). Thyroglobulin (669 kDa), ferritin (440 kDa), catalase (232 kDa), lactate dehydrogenase (142 kDa), and bovine albumin (66 kDa) (Amersham Biosciences, Piscataway, NJ, USA) were used as molecular weight standards. For lectin separation, electrophoresis was performed at 45 mA, 180 V, and 4 °C, and the separated bands were visualized by silver staining.

### 4.9. Carbohydrate and Metal Ion Content Determination

Carbohydrate content was determined according to the sulfuric phenol method proposed by Dubois et al. [74], using glucose as the standard. For this purpose, lectin was dissolved in NaCl (0.15%), dialyzed against EDTA solution (0.02 M) in NaCl (15 mM) for 12 h, and then digested with nitric acid (38%). Metal ion content was determined by plasma spectrophotometry (ICP/OES, Perkin-Elmer Optima™ 8300, Waltham, MA, USA) using a standard calibration curve for each ion (Ca^+2^, Mg^+2^, Mn^+2^, Cu^−1^, and Zn^+2^), whereby their respective concentrations in the lectin were calculated by graphic interpolation [6,9].

### 4.10. Effect of Metal Ions on Hemagglutination

Prior to assessing the influence of metal ions on hemagglutination, the studied lectins were demetallized by dialysis against EDTA (2 mM) in NaCl (15 mM) for 12 h, and the titer was determined as described above. To determine the effect of metals on agglutination, 96-well (U-shaped) microplates were used, and 50 µL of PBS, 50 µL of demetallized lectin, and 50 µL of a solution (0.5 mM) containing metal ions (CaCl_2_, MgCl_2_ and MnCl_2_) were added to each well. After allowing the reaction to incubate for 60 min at room temperature, 50 µL of the O erythrocyte suspension was added, and the agglutination titer was observed [6,9].

### 4.11. Influence of pH and Temperature on Hemagglutination

For assessing the influence of pH and temperature on hemagglutination, lectins were dissolved in PBS (1 mg/mL) and were incubated for 60 min at room temperature with the following buffers (2 mM): sodium acetate (pH 3. 5, 4.0, 4.0, 4.5, 5.0, and 5.5), sodium phosphate (pH 6.0, 6.5, 7.0, and 7.5), tris-HCl (pH 8.0 and 8.5), and glycine-NaOH (pH 9.0, 9.5, 10.0, and 10.5), all of which were purchased from Sigma Chemical Co. (St Louis, MO, USA). Subsequently, each solution was dialyzed against two fresh PBS volumes for 12 h at 4 °C, and the hemagglutination tests were performed using trypsinized human erythrocyte O [13].

For this purpose, lectins were dissolved in PBS (1 mg/mL) and heated in a water bath for 30 min at 25, 30, 40, 40, 50, 50, 60, 60, 70, 80, 90, and 100 °C, after which the samples were cooled on ice. Finally, the hemagglutination test was performed using trypsinized human erythrocyte O [13].

### 4.12. Statistical Analysis

The reported study findings are based as the average of three replicates (mean ± standard deviation). Moreover, both one way analysis of variance (ANOVA) and Tuk-ey’s test were conducted to determine the differences between samples using the commercial statistical program GRAPHPAD INSTAT (San Diego, CA, USA) version 3.0.

## 5. Conclusions

In this work we report the partial characterization of two black *Phaseolus vulgaria* lectins, BBV and BBS, which are tetrameric glycoproteins with subunits of 27.34 and 28.17 kDa, respectively. Both lectins exhibited capacity to hemagglutinate human and animal erythrocytes, which was inhibited by fetuin and required metal ions to carry out their biological activity, such as Mg and Ca, for which temperatures below 60 °C and conditions close to neutral (LBBV) and alkaline (LBBS) pH were found to be the most optimal. Furthermore, the difference in the observed characteristics was attributed to the structure of these proteins, the seed variety involved, and the agronomic conditions under which these plants were grown. More advanced analyses are needed to have a complete characterization of these lectins Future research may be oriented to the knowledge of the proteomics of these lectins, such as sequencing, Malditof, and oligosaccharide identification, among others.

## Figures and Tables

**Table 1:** Purification of black bean lectins on immobilized stroma.

Fraction	Protein Concentration (mg/mL)	Hemagglutination Titer * (Units)	Specific Activity (HA)	Purification Factor	Lectin (%)
BBV					
Crude extract	1086.47	16	0.0147	1	
Protein bound to stroma	0.0488	8	163.93	11,151.7	0.0045 ^a^
BBS					
Crude extract	1034.77	1.09 × 10^12^	1.05 × 10^9^	1	
Protein bound to stroma	0.1062	8.38 × 10^6^	7.89 × 10^7^	0.075	0.0103 ^b^

Data shown represent mean ± SEM obtained in at least three independent experiments. Means with different superscripts are significantly different (Tukey’s test, *p* < 0.05). * Tripsinized erythrocytes type O

**Table 2:** Hemagglutination activity of purified lectin from both black bean varieties (surco and vara) on trypsinized human and animal erythrocytes.

Erythrocyte Type	Lectins	
Human	BBV	BBS
	Hemagglutination Titer	
A	16 ± 0 ^a^	2097152 ± 0 ^a^
B	16 ± 0 ^a^	67108864 ± 0 ^b^
O	4 ± 0 ^b^	4.39 × 10^12^ ± 0 ^c^
Animals		
Rabbit	128 ± 0 ^a^	134217728 ± 0 ^a^
Chicken	32 ± 0 ^b^	262144 ± 0 ^b^
Hamster	1.12 × 10^15^ ± 0 ^c^	7.92 × 10^28^ ± 0 ^c^
Bull	ND	1024 ± 0 ^d^
Pig	16 ± 0 ^d^	524288 ± 0 ^e^
Sheep	2 ± 0 ^e^	8192 ± 0 ^f^

Data shown represent mean ± SEM obtained in at least three independent experiments. Means with different superscripts are significantly different (Tukey’s test, *p* < 0.05).

**Table 3:** Metal content in BBS and BBV lectins.

Metal	Native Lectins		Demetallized Lectins	
	BBV	BBS	BBV	BBS
	Concentration (ppm)			
Ca^+2^	597.5 ± 0.03 ^a^	823.0 ± 0.05 ^a^	284.0 ± 0.01 ^b^	199.0 ± 0.02 ^b^
Cu^−1^	274.5 ± 0.02 ^a^	212.5 ± 0.01 ^a^	185.0 ± 0.01 ^b^	186.0 ± 0.02 ^b^
Mg^+2^	487.0 ± 0.03 ^a^	774.5 ± 0.25 ^a^	358.0 ± 0.02 ^b^	171.0 ± 0.06 ^b^
Mn^+2^	224.0 ± 0.01 ^a^	176.5 ± 0.02 ^a^	185.0 ± 0.01 ^b^	170.0 ± 0.02 ^a^
Zn^+2^	306.5 ± 0.02 ^a^	316.5 ± 0.08 ^a^	182.5 ± 0.01 ^b^	182.0 ± 0.02 ^b^

Data shown represent mean ± SEM based on the results obtained in at least three independent experiments. Means with different superscripts are significantly different between lectins (Tukey’s test, *p* < 0.05).

**Table 4:** Hemagglutination activity of two black bean lectins after demetallization and ion metal addition.

	Native Lectin	Demetallized Lectin	Demetallized Lectins Reconstituted with Metals (Hemagglutination Titer)		
	Hemagglutination Titer		Ca^2+^	Mg^2+^	Mn^2+^
BBV	2 ± 0 ^a^	0 ± 0 ^a^	128 ± 0 ^a^	4096 ± 0 ^a^	4 ± 0 ^a^
BBS	1,073,741,824 ± 0 ^b^	16 ± 0 ^b^	6.87 × 10^10^ ± 0 ^b^	1.09 × 10^12^ ± 0 ^b^	2048 ± 0 ^b^

Data shown represent mean ± SEM based on the results obtained in at least three independent experiments. Means with different superscripts are significantly different between lectins (Tukey’s test, *p* < 0.05).

**Table 5:** Effects of different carbohydrates on the LBBV and LBBS hemagglutination activity.

	Inhibitory Concentration (mg/mL) *	
Monosaccharides	LBBV	LBBS
Glucose	ND	ND
Galactose	ND	ND
Mannose	ND	45.04
Fructose	ND	ND
Oligosaccharides		
Maltose	ND	ND
Trehalose	ND	ND
Raffinose	252.21	ND
Glycoproteins		
Ovoalbumin	ND	ND
Fetuin	0.0075	0.129

Notes: Tripsinized erythrocytes type O, * Lowest concentration resulting in complete inhibition, Carbohydrate concentration 0.5 M, Lectin concentration 0.1 mg/mL. Data shown represent mean ± SEM based on the results obtained in at least three independent experiments.

## Data Availability

Not applicable.
